# Pregnancy in Woman with Kawasaki Disease and Multiple Coronary Artery Aneurysms

**DOI:** 10.5935/abc.20170185

**Published:** 2018-01

**Authors:** Walkiria Samuel Avila, Antônio Fernando Diniz Freire, Alexandre Anderson de Sousa Soares, Ana Neri Rodrigues Epitácio Pereira, José Carlos Nicolau

**Affiliations:** Instituto do Coração (InCor) - Hospital das Clínicas da Faculdade de Medicina da Universidade de São Paulo (HCFMUSP), São Paulo, SP - Brazil

**Keywords:** Pregnancy, Mucocutaneous Lymph Node Syndrome, Coronary Aneurysm

## Introduction

Kawasaki disease (KD), first described in 1967, is a systemic vasculitis of unknown cause.^1^ It is an important cause of cardiac diseases in children aged younger than five years.^2^ KD is an autoimmune disorder whose clinical features include high fever, exanthema, conjunctivitis, cervical linfadenopathy and peripheral edema. Laboratory tests are compatible with acute inflammatory condition.

KD predominantly affects the coronary arteries, which is the most important clinical manifestation of the disease, varying from dilation and stenosis to aneurysm (incidence of 5% in patients with adequate treatment, and 25% in untreated patients). Giant coronary artery aneurysms (CAAs), i.e., CAAs with diameters > 8 mm, are associated with increased risk of thrombosis, acute myocardial infarction (AMI) and sudden death.^2^

Lack of diagnosis and treatment of KD at its acute phase during childhood contributes to increased prevalence of pregnant women with vascular sequelae of KD.^3^ Management of these patients has not been established, especially in symptomatic patients and, in existing literature, premature labor has been performed in these cases. There are no Brazilian reports on the theme, which are more commonly found in the American and Japanese literature.

The aim of this study is to describe a successful management of a patient with giant CAA, sequela of KD with thrombotic complication, from the first trimester of pregnancy until term birth.

### Case report

**Figure 1:**
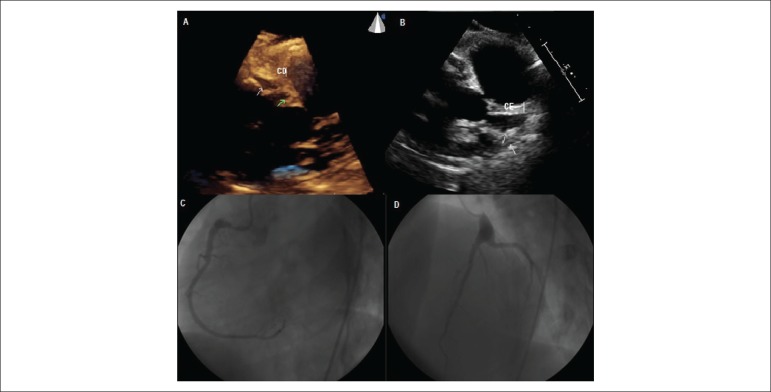
(A and B) Echocardiography: ejection fraction 68%; left atrium 35 mm; septum 8mm; posterior wall 8mm; left ventricular diastolic diameter 45mm; left ventricular systolic diameter 30 mm; PSAP 40 mmHg. Dilatation of left coronary artery (7 mm). Left ventricle with preserved systolic function and myocardial thickness, with no changes in segmental wall motion. (C and D) Cardiac catheterization (10/2013): coronary artery ectasia. Dominant coronary with 50% proximal, eccentric tubular lesion and coronary thrombosis; left coronary artery trunk with aneurysmatic dilatation at the distal third; anterior descending artery with ectasia at the proximal third, without obstructive lesions. Circumflex artery with proximal ectasia, without obstructive lesions.

**Figure 2:**
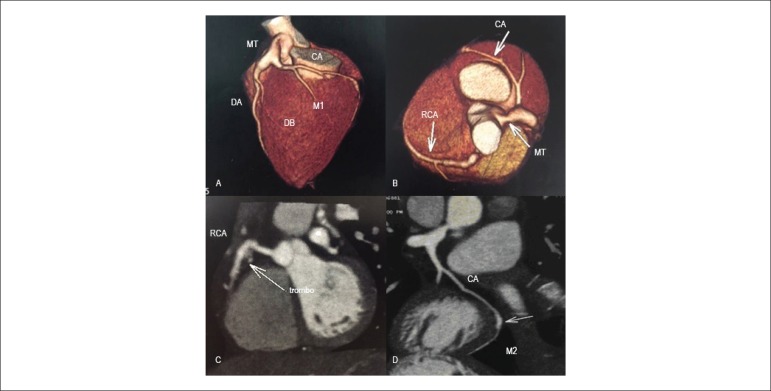
Coronary computed tomography angiography showed aneurysm at the distal third of the left coronary artery trunk (9mm) and anterior descending artery ostium (9.5 mm); circumflex artery with ectasia at the ostium (3.7 mm); marginal branch 2 with aneurysm at the distal segment; right coronary artery with saccular aneurysm (9.8 mm at the greatest diameter), mural thrombus and small regions of calcification. Total score of 23.81 (Agaston) and 38.59 (Volume). MT: main trunk of the left coronary artery; DA: anterior descending artery; DB: diagonal branch; CA: circumflex artery; M1: left marginal artery; M2: second marginal branch of circumflex artery; RCA: right coronary artery; LA: left atrium; Ao: aorta

A 32-year old patient was admitted to the emergency care at week nine of first pregnancy, with dyspnea and slight, precordial pain during great efforts, of short duration and well tolerated. The patient had a history of ST-elevation myocardial infarction of inferior wall at the age of 30, with giant aneurysms and coronary artery thrombosis evidenced by coronary angiography (Figure 1C and 1D). The patient underwent coronary computed tomography angiography, which confirmed the previous findings, with evidence of coronary artery ectasia with multiple aneurysmatic dilatations and mural thrombus (Figure 2). The patient received the diagnosis of KD and was referred to outpatient follow-up. At the occasion, the patient was using simvastatin, clopidogrel, atenolol and acetylsalicylic acid (ASA). At physical examination on admission, the patient was eupneic, with blood pressure of 110/60 mmHg, heart rate of 80 bpm, normal heart sounds without murmurs, normal pulmonary auscultation, abdomen free of abnormal signs, and normal pulse rate. Regarding complementary tests, electrocardiography showed sinus rhythm with diffuse ventricular repolarization; transthoracic echocardiogram is depicted in Figure 1A and 1B.

Despite recommendations received in outpatient visits on contraindications for pregnancy, the patient got pregnant, and a close monitoring of the patient was started. A daily dose of 100 mg of ASA, 60 mg of propranolol and 40 mg of enoxaparin were prescribed, and routine obstetric exams showed normal fetal vital signs. After 29 weeks of pregnancy, the patient had progressive worsening of cardiac function to New York Heart Association (NYHA) class III, with daily, atypical palpitations, which caused the patient to get a sick leave to rest at home.

During the week 34, the patient had diffuse chest pain, dyspnea and uterine contractions. The patient was then hospitalized for a rest and adjustment of medication. Obstetric examination revealed irregular, weak contractions, fundal height of 33 cm, impenetrable cervix, a single fetus in longitudinal position, cephalic presentation, regular heart beat at 128 bpm. Fetal assessment was performed by fetal biophysical profile and normal doppler velocimetry of umbilical arteries. Estimated fetal weight was adequate (percentile of Hadlock growth curve). There was a marked improvement in clinical and obstetric conditions as result of adjustments in propranolol (80 mg/day orally) and enoxaparin (60 mg/2x day subcutaneously) doses, as well as administration of sublingual nitrate (only if needed) and vaginal tablets of natural micronized progesterone (200 mg/2x day). There were no changes in electrocardiographic or echocardiographic patterns during hospitalization. At week 37, cesarean section and tubal ligation were indicated. The procedure was successfully performed by the Central Institute obstetric staff at the Heart Institute (InCor) of the General Hospital of the University of Sao Paulo Medical School. Newborn was born healthy, weight 2,860 g, appropriate for gestational age, Apgar score of 9 and 10 at fifth and tenth minute of life, respectively. Tubal ligation was performed following delivery, with previous consent of the spouse. Enoxaparin was discontinued 12 hours before and restarted 24 hours after cesarean section. Forty-eight hours later, warfarin was prescribed and readjusted until the prothrombin international normalized ratio (INR) was 2; at this time, enoxaparin was suspended and the patient was discharged. At the clinical visit 60 days thereafter, the patient was asymptomatic, breast feeding and using warfarin (INR = 2) and ASA (100 mg/d).

## Discussion

In the present case, strategies for prevention of complications of giant CAAs secondary to KD and acute infarction were successful in terms of maternal-fetal health.

Preventive therapy was planned during patient’s first medical visit at week 9 of pregnancy. The strategy consisting of outpatient follow-up, hospitalization and delivery with interventional support at a cardiology hospital was chosen because of patient instability. However, such procedure is not considered routine in the literature in symptomatic patients.^3^

We also considered the influence of the hyperkinetic, hypercoagulable state of pregnancy on the occurrence of expected complications (thrombosis, myocardial infarction and sudden death) in this patient. The potential risk of arterial rupture and/or dissection is increased with presumed arterial changes including fragmentation of reticular fibers, decrease in mucopolysaccharide content and loss of normal elastic fiber structure.^4^

In the study by Wei et al.,^5^ that included 38 cases of KD, thrombosis was seen in 17 patients, which has been hypothesized to be caused by insufficient anticoagulant therapy. In a meta-analysis including 159 children with giant CAA, Su et al.^6^ reported that coronary occlusion, AMI and death were significantly lower in children treated with warfarin plus aspirin than in those treated with aspirin alone. In this line of thought, the progressive activation of coagulation factors in the second half of pregnancy, and the maximum activation at delivery made the authors recommend anticoagulation with dose adjustment combined with ASA. Enoxaparin was used in place of warfarin during pregnancy due to risk of hemorrhage and fetal toxicity, in prophylactic dose until week 34 and then therapeutic dose until 12 hours before delivery. The drug was then restarted until warfarin was reintroduced for maintenance of INR within target range.

The history of myocardial infarction increased the pregnancy risk, although ventricular function was preserved and was favorable to patient’s progression. Increased myocardial metabolic demand, due to increased cardiac output and oxygen consumption related to pregnancy, was the cause of frequent complaints of angina and dyspnea, which were controlled by propranolol. At 60 mg/day, the drug did not affect fetal growth until week 32 of pregnancy. Arterial hypotension, resulting from a decrease in peripheral vascular resistance, limited the use of nitrates for the supposed risk of decreased uteroplacental blood flow.

During the third trimester of pregnancy, high-amplitude uterine contractions (Braxton Hicks) become more frequent and may be confused with premature labor, accounting for 75% of births before week 37 of pregnancy.^7^ These contractions cause oscillations in venous return and in heart rate, and may cause instability in women with limited cardiac reserve, which was the cause of hospitalization of the patient in the week 32. Together with the obstetrician, a decision was made to not anticipate delivery, adjust medication for control of clinical obstetric symptoms until fetal maturity was reached.

With respect to the type of delivery chosen, a study on 13 women with KD^8^ and coronary artery lesions, showed that vaginal deliveries under epidural anaesthesia in 9 patients, and caesarean section was performed in 3 symptomatic patients. These data corroborate the clinical decisions made in this case. Also, tubal ligation was chosen as the safest contraceptive method due to contraindications of a new pregnancy.

## Conclusion

This report added to the literature one case of successful term pregnancy in a symptomatic patient with multiple CAAs secondary to KD and history of myocardial infarction. The study illustrated the importance of the multidisciplinary approach to reach the full-term of a high-risk pregnancy. However, family planning, including counseling on genetics and possibility of a new pregnancy, is still essential. The risk of complications cannot be neglected regardless of the therapeutic strategy adopted.
